# Elevated p38MAPK activity promotes neural stem cell aging

**DOI:** 10.18632/aging.102994

**Published:** 2020-04-03

**Authors:** Leire Moreno-Cugnon, Olatz Arrizabalaga, Irantzu Llarena, Ander Matheu

**Affiliations:** 1 Biodonostia Health Research Institute, Group of Cellular Oncology, San Sebastian, Spain; 2 Optical Spectroscopy Platform, CIC biomaGUNE, Basque Research and Technology Alliance (BRTA), San Sebastian, Spain; 3 CIBERfes, Madrid, Spain; 4 IKERBASQUE Basque Foundation for Science, Bilbao, Spain

**Keywords:** p38MAPK, aging, neural stem cell, pharmacological inhibition

## Abstract

Age-progressive neural stem cell (NSC) dysfunction leads to impaired neurogenesis, cognitive decline and the onset of age-related neurodegenerative pathologies. p38MAPK signalling pathway limits stem cell activity during aging in several tissues. Its role in NSCs remains controversial. In this work, we show that p38MAPK activity increases in NSCs with age in the subventricular zone (SVZ) and its pharmacological inhibition is sufficient to rejuvenate their activity *in vitro*. These data reveal a cell-autonomous role for p38MAPK increase in decreasing NSC homeostasis with age. This information shed light in the role of p38MAPK in NSC aging.

## INTRODUCTION

Adult stem cells are responsible for the maintenance of tissue homeostasis during the life of the organism and this control is due to their specific characteristics of quiescence, self-renewal potential and capacity to differentiate to specialized cell lineages. Decline in stem cell function with age has been described in several compartments including muscle, brain, intestine, skin, and the hematopoietic system in mammals, and it is linked to decreased organ function and degenerative processes [1]. Stem cell aging is promoted by deregulation in different cell-intrinsic pathways, in cell-extrinsic signals that maintains microenvironment and niche homeostasis, as well as by systemic factors [1, 2]. The identification of critical underlying mechanisms responsible for the maintenance of the function of the adult stem cells or the delay of their exhaustion would allow delaying the tissue deterioration associated with age, an idea with great therapeutic potential.

p38 mitogen-activated protein kinase (p38MAPK) is a relevant sensor of multiple types of intrinsic and extrinsic stresses and controls key processes of cell homeostasis such as proliferation, death, self-renewal and differentiation [3]. The activation of p38MAPK signaling triggers the exhaustion of stem cells in different compartments, such as hematopoietic [4] lung [5] or muscle [6, 7]. In the brain, the activity of p38MAPK plays regulatory roles during embryo development and postnatal stage in neural stem/progenitor cells (NSC). However, the results obtained so far are disparate and show both positive and negative effects of p38MAPK signaling in NSC function [8–14].

NSCs reside in the subventricular zone (SVZ) of the lateral ventricle and the subgranular zone of the dentate gyrus (DG) in the hippocampus in the adult mammalian brain. NSCs give rise to intermediate progenitor cells, which divide generating immature neurons, that subsequently integrate into the neural networks within the neurons incorporated in the olfactory bulb or DG, respectively. The new neurons that incorporate into the neuronal circuitry are responsible for olfactory ability, memory, learning and behavior [15]. With age, there is a decline in functional NSCs and this correlates with the presence of lower neurogenesis, and the diminishment in the production of new neurons limit plasticity and repair of the brain and underlies age-related cognitive decline [16, 17]. In this work we characterized the impact of p38MAPK in NSCs in SVZ.

## RESULTS AND DISCUSSION

**Figure 1:**
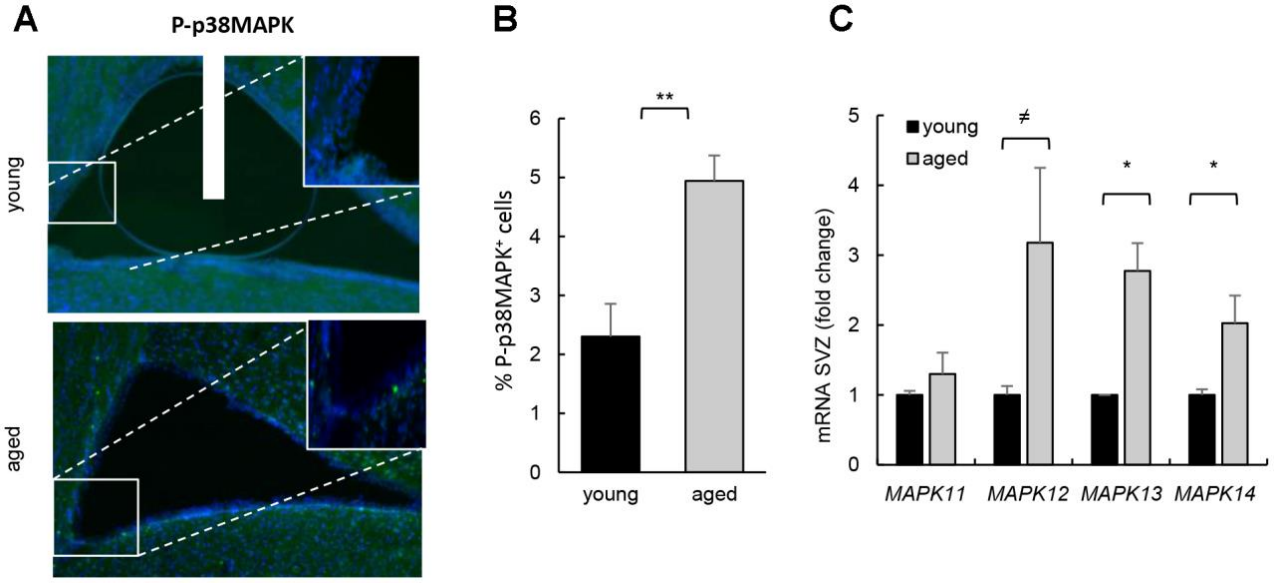
**Increased p38MAPK activity in SVZ neurogenic niche with aging.** (**A**) Representative immunofluorescence for P-p38MAPK in SVZ of young (2 month-old) and aged (over 24 month-old) *C57BL/6J* mice (n≥2). (**B**) Quantification of number of P-p38MAPK positive cells in this region. (**C**) *MAPK11, MAPK12, MAPK13* and *MAPK14* mRNA levels in SVZ of young (2 month-old) and aged (over 24 month-old) *C57BL/6J* mice (n≥4).

We first determined the activity of p38MAPK in young (2 month-old) and aged (≥ 2 year-old) SVZ from *C57BL/6J* mice. Immunofluorescence showed that p38MAPK phosphorylated in the activating residues (P-p38MAPK) was low in the cells along the SVZ in young mice and significantly increased in over 2 year-old animals (Figure 1A, 1B). Moreover, the expression of all p38MAPK family members (*MAPK11 (β), MAPK12 (γ), MAPK13 (δ)* and *MAPK14 (α)*) was elevated in *ex vivo* SVZ cells from aged compared to young mice (Figure 1C). A recent study detected diminishment of P-p38MAPK and total p38MAPK with age, particularly in the DG but also in SVZ [13]. In this study, younger animals (3 weeks *vs* 1.5 year-old mice) were analyzed than in ours (2 months *vs* over 2 year-old). Since it has been shown that the expression of total and phosphorylated p38MAPK decreases during embryo development and, at least, up to postnatal day14, where levels are undetectable [10], the differences between studies might be explained by the different age of the animals.

**Figure 2:**
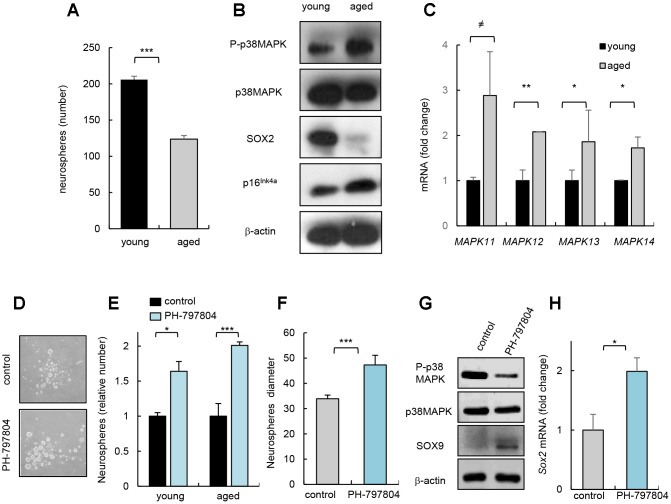
**p38MAPK activity regulates NSC/progenitor aging *in vitro*.** (**A**) Quantification of neurospheres from young (2 month-old) and aged (over 24 month-old) *C57BL/6J* mice (n=3). (**B**) P-p38MAPK, SOX2 and p16^Ink4a^ expression in neurospheres derived from animals at the indicated ages (n=3). (**C**) Analysis of MAPK isoforms in neurospheres. (**D**) Representative image and (**E**) quantification of neurospheres derived from the SVZ of young and aged *C57BL/6J* mice treated with p38MAPK inhibitor (PH-797804) or control (DMSO) (n=4). (**F**) Quantification of the diameter of secondary neurospheres derived from aged mice treated with PH-797804 or control (n=4). (**G**) Representative western blot of P-p38MAPK, p38MAPK, SOX9 and ß-actin in 2^ry^ neurospheres from aged mice (n=2). (**H**) Quantification of *Sox2* mRNA levels in aged cells (n=3).

Next, we cultured neurospheres harvested from SVZ area of mouse of different ages (2 month-old *vs* 2 years) and observed, confirming previous studies, that aged cells presented decreased capability of neurosphere formation (Figure 2A), which correlated with lower levels of SOX2 stem cell regulator and higher p16^Ink4a^ expression, gene related to cell cycle and senescence (Figure 2B). Interestingly, neurospheres derived from aged mice contained higher levels of P-p38MAPK (Figure 2B) and these cells also showed higher mRNA levels of all p38MAPK family members (Figure 2C). Together, our data show that the increase in p38MAPK activity coincides with the decline in the activity of NSCs *in vitro* and *in vivo*. On the contrary, the previous cited study detected decreased *p38* mRNA expression, and lower p38α and P-p38MAPK immunoreactivity in SVZ neurospheres isolated from 6 month-old mice compared to 6 weeks old [13].

Previous studies observed that pharmacological inhibition of p38MAPK in NSC/progenitors derived from embryos or up to 4 month-old adult mice protects against apoptosis [8], increases proliferation [10], enhances self-renewal and differentiation potential [9, 18], and promotes migration [11] *in vitro*. In contrast, 6 month-old *p38α* conditional knockout mice under the control of the *Nestin* gene promoter formed lower and smaller number of neurospheres than controls *in vitro* and reduced the proliferation of progenitors *in vivo* [13]. Next, we tested whether inhibition of p38MAPK could prevent NSC/progenitor aging. For this, we cultured cells from SVZ of young and aged mice with PH-797804, a selective p38MAPK inhibitor [19]. We found that young and aged cells incubated with the p38MAPK inhibitor PH-797804 formed higher number of neurospheres in both ages (Figure 2D, 2E). The elevation in neurosphere formation ability of aged cells correlated with a larger size (Figure 2F), and a a reduction in P-p38MAPK as well as higher SOX9 and *Sox2* levels (Figure 2G, 2H), supporting that decreased p38MAPK rejuvenates aged NSC/progenitor function.

**Figure 3:**
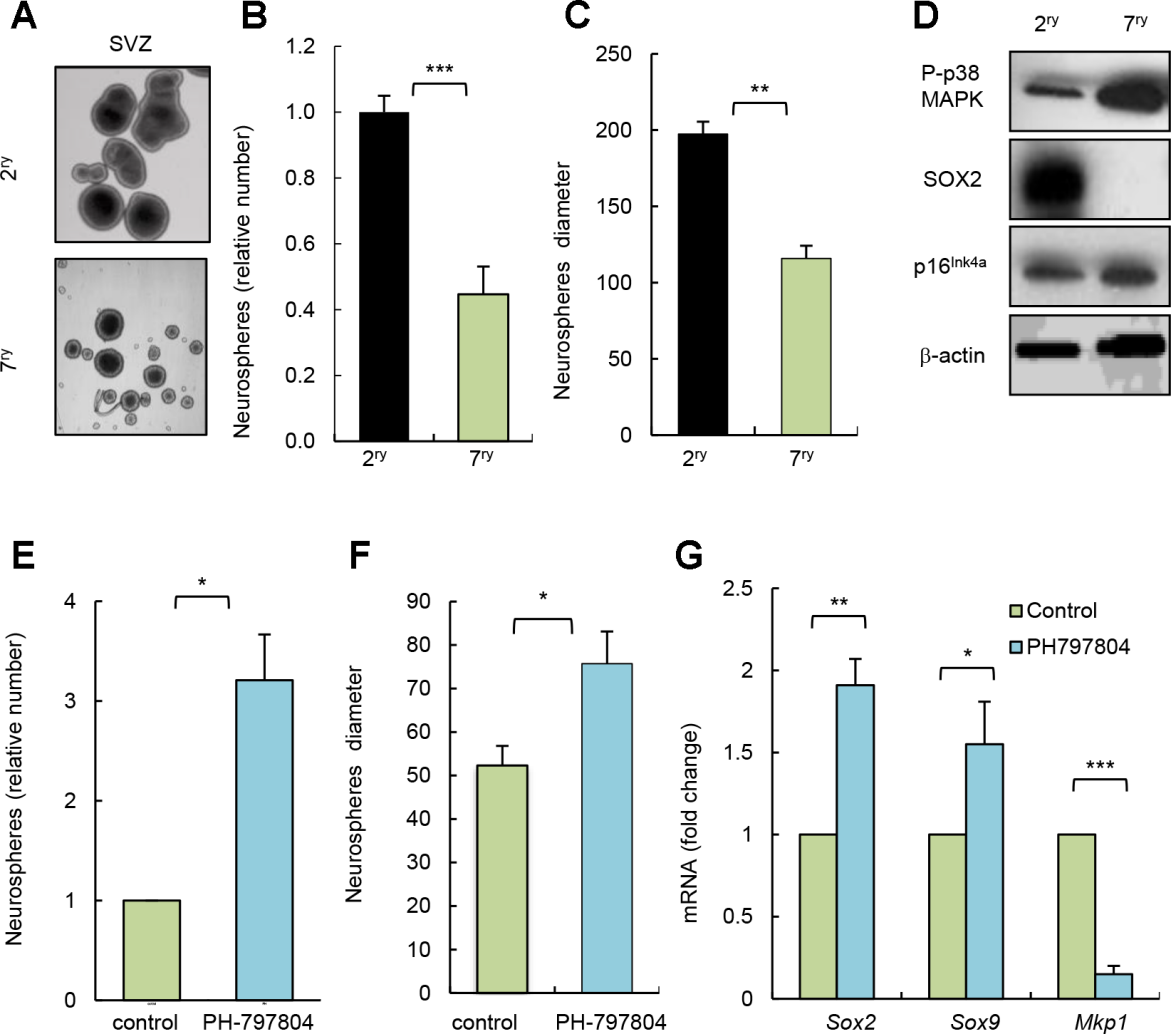
**Pharmacological inhibition of p38MAPK rejuvenates NSC function *in vitro*.** (**A**) Representative image and (**B**, **C**) quantification of number of neurospheres and their diameter at passage 2 (2^ry^) and 7 (7^ry^) from SVZ *C57BL/6J* mice (n=3). (**D**) Representative western blot of P-p38MAPK, SOX2, p16^Ink4a^ and ß-actin at indicated conditions (n=3). (**E**) Relative number of neurospheres formed from passage 7 cells treated with p38MAPK inhibitor (PH-797804) or control (DMSO) (n=3). (**F**) Quantification of the diameter of neurospheres from 7^ry^ passage treated with PH-797804 or control (n=3). (**G**) *Sox2*, *Sox9* and *Mkp1* expression in neurospheres maintained for passages (n=3).

Long term-cultured cells *in vitro* share multiple characteristics of physiological aging [20]. To further test the impact of p38MAPK inhibition in NSC/progenitor aging, we serially passaged neurosphere cultures. Cells after 7 passages (7^ry^) generated smaller and lower number of neurospheres than after passage 2 (2^ry^) (Figure 3A–3C). This correlated at molecular level with decreased SOX2 and increased p16^Ink4a^ expression (Figure 3D). In this context, cells from 7^ry^ passage also displayed increased P-p38MAPK (Figure 3D). Moreover, 7^ry^ cells treated with p38MAPK inhibitor formed 3 times more neurospheres than non-treated control cells (Figure 3E). Treatment with the inhibitor also promoted a significant shift to larger size of neurospheres (Figure 3F) and elevation of *Sox2* and *Sox9* stem cell genes, and decrease of *Mkp1/Dusp1* differentiation marker [21] (Figure 3G). These results further show that inhibition of intrinsic p38MAPK activity restores NSC activity and, together with the above-indicated studies, highlight the relevance of p38MAPK signaling in NSC homeostasis. In line with this idea, single cell transcriptomic analysis revealed that MAPK cascade related genes are altered in NSC/progenitor subpopulations with aging [22]. Moreover, our results also indicate that the role of p38MAPK in NSC/progenitor activity and aging, in contrast to stem cell populations in other tissues, might be more complex and may depend on a fine regulation of the timing and levels of its activity, the interactions within the neurogenic niche [14], and also with other signaling pathways. In this regard, Wnt pathway might be involved [13]. Moreover, EGF signaling might be associated with p38MAPK, since p38MAPK function is a crucial mediator of this pathway, and it is well known that EGF promotes NSC proliferation *in vitro* and *in vivo* [23] and decreased EGF receptor signaling associates with aging in the SVZ niche [24].

In summary, we show that p38MAPK activity is elevated in SVZ neurogenic niche of aged mice *in vivo* and in neurospheres derived from aged cells *in vitro*. Moreover, pharmacological inhibition of p38MAPK in aged neurospheres rejuvenates NSC/progenitor activity *in vitro*. Ultimately, our data postulate p38MAPK activity as an intrinsic regulator of NSC aging.

## MATERIALS AND METHODS

### Mice handling and ethics statement

The *C57BL/6J* (Jackson Laboratory) mice were housed in specific pathogen-free barrier areas of the Biodonostia Health Research Institute. Mice were maintained and handled in compliance with the animal research regulations specified in the European Communities Directive [2010/63/EU]. All Animal studies were approved by the Biodonostia Health Research Institute Animal Care Committee.

### Tissue immunofluorescence

Coronal serial sections of 50 μm were collected via SM2010 R Sliding Microtome (Leica), and selected brain sections were blocked with 10% donkey serum and incubated with anti-P-p38MAPK (1:200; rabbit, Cell Signalling) overnight at 4 °C. Nuclei were stained with DAPI (Sigma). Images were acquired with an inverted confocal light scanning microscope (CLSM 510Meta, Zeiss) with a 63x objective (NA 1.3, aprochromat) in sequential mode with 1024 x 1024 scan size. Processing and analysis was performed on the maximal intensity projection of the z-stack, and selection of the area was accomplished using the nucleus positives areas. Fiji public domain, open source software was used.

### RNA analysis

Total RNA was extracted with Trizol (Life Technologies). Reverse transcription was performed using random priming and Superscript Reverse Transcriptase (Life Technologies), according to the manufacturer’s guidelines. Quantitative real-time PCR was performed using Absolute SYBR Green mix (Thermo Scientific) in an ABI PRISM 7500 thermocycler (Applied Biosystems).

### Western blot analysis

Immunoblots were performed following standard procedures. Equal amounts of protein (20 μg) were separated on 15% SDS polyacrylamide gels and blotted onto nitrocellulose membranes (BioRad). Primary antibodies were SOX9 (Millipore), SOX2 (Millipore), P-p38MAPK (Cell Signalling), total p38MAPK (Santa Cruz), p16^Ink4a^ (Santa Cruz) and β-actin (Sigma). Secondary antibodies were HRP-linked anti-mouse or rabbit (DAKO). Detection was performed by chemiluminescence using ECL (Amersham).

### Neurosphere cultures

Isolation, culture, and assays of NSCs were carried out as previously described [25]. Briefly, NSCs were isolated from the mouse SVZ and grown for 10 days in DMEM/F12 growth medium (Sigma) in the presence of EGF (20 ng/mL, Sigma) and FGF-2 (20 ng/mL, Gibco). Primary neurospheres after being counted, were treated with accutase (Sigma) for 5 min, mechanically dissociated to a single-cell suspension and re-plated in growth medium containing EGF and FGF for 10 days (secondary neurospheres, 2^ry^). Serial passage experiment was done repeating this methodology up to seven passages (7^ry^ neurospheres).

### Statistics

Data are presented as mean values ± S.E.M., with the number of experiments (n) in parentheses. Unless otherwise indicated, statistical significance (p-values) was calculated using the Student’s t -test. Asterisks (*, **, and ***) indicate statistical significance (p < 0.05, p < 0.01, and p < 0.001, respectively).
